# A scoping review of squeeze pouch use among infants and children: Frequency, types, sociodemographic characteristics and parental experiences

**DOI:** 10.1111/1747-0080.70030

**Published:** 2025-07-24

**Authors:** Bianca Smith, Catharine A. K. Fleming, Ami Seivwright, Katherine Kent

**Affiliations:** ^1^ School of Medical, Indigenous and Health Sciences University of Wollongong Wollongong Australia; ^2^ School of Health Sciences Western Sydney University Sydney Australia; ^3^ Monash Climate Change Communication Research Hub Monash University Melbourne Australia

**Keywords:** child feeding practices, infant nutrition, nutritional quality, scoping review, squeeze pouches

## Abstract

**Aims:**

Demand for infant and child foods in squeeze pouches is increasing, yet limited evidence exists to inform public health strategies on who uses them and why. The aim of this scoping review was to explore the frequency and types of squeeze pouches consumed by children, the sociodemographic characteristics of users and insights from parental experiences regarding their use.

**Methods:**

A scoping review was conducted in accordance with the Joanna Briggs Institute guidelines for scoping reviews. Between January and April 2024, three databases were searched (MEDLINE, Scopus and CINAHL) in addition to grey literature. Two independent reviewers screened 125 articles, of which 16 underwent full‐text review. Results were summarised narratively.

**Results:**

Eleven studies across five countries were included. Eight studies reported on the prevalence of squeeze pouch consumption, ranging from 23.5% to 82.8% for infants and children. Frequency of intake ranged from daily (*n* = 4; 8.7%–29.2%), weekly (*n* = 7; 20.9%–75.2%), to monthly consumption (*n* = 7 16.7%–70.4%), depending on the study. Predominantly fruit‐ and dairy‐based pouches were consumed. Frequent use was linked to higher deprivation, childcare use, larger families, early breastfeeding cessation and early solid food introduction. Studies on parental perceptions (*n* = 6) reported their perceived convenience, healthiness and low cost and concerns related to waste.

**Conclusions:**

This review identified widespread use of squeeze pouches among infants and children, particularly fruit and dairy‐based pouches. More research is needed on their nutritional impact to guide public health strategies promoting healthy early feeding practices.

## 1 INTRODUCTION

The first 1000 days of an infant's life represent a critical foundational period in which the confluence of epigenetics, biological determinants and environmental influences such as nutrition profoundly impact an infant's health and development.^1^, ^2^, ^3^ The World Health Organisation (WHO) recommends that infants be exclusively breastfed for the first 6 months of life, followed by the introduction of age‐appropriate complementary foods alongside continued breastfeeding up to 2 years of age or beyond to meet increasing nutritional demands.^4^ Beyond infancy, optimal nutrition remains essential throughout childhood and adolescence to support rapid growth and cognitive development.^5^ Malnutrition and high Body Mass Index (BMI) can impact health at all life stages, including infancy, childhood and adolescence, with long‐term consequences for growth, physical maturation and neurological development.^6^, ^7^ Establishing healthy eating behaviours from infancy through adolescence is important, as dietary patterns formed during these periods can influence lifelong health outcomes.^8^, ^9^

Navigating this time can be challenging for parents, with stress about feeding their child, concerns about growth and conflicting information regarding complementary feeding,^10^, ^11^ providing manufacturers an opportunity to capitalise.^5^, ^12^, ^13^ In recent decades, a significant shift in feeding practices has emerged following the introduction of foods in squeeze pouches in 2008.^3^ These products have experienced significant market growth and now dominate commercial infant and toddler food markets globally.^1^, ^9^, ^14^, ^15^ Recent audits have also highlighted their poor nutritional composition. For example, international analyses of the nutritional composition of squeeze pouches reported that they were consistently high in added sugars^3^, ^16^, ^17^ and low in iron.^18^ It is possible that among the current cost‐of‐living crisis, which is disproportionately affecting the cost of fruits and vegetables,^19^ the affordability of these products may displace more expensive fresh produce.^20^ Originally designed for infants, concerningly, the market share of squeeze pouches has also expanded beyond infants, now targeting toddlers, preschoolers, school age children older and even adults.^21^ There is limited research on how prolonged squeeze pouch consumption influences dietary habits in older children. Some concerns have been raised that their widespread use could displace more diverse, nutrient‐rich foods, but further research is needed to understand their role in children's overall diets.^1^, ^16^, ^17^

Given these concerns, expert recommendations advise that squeeze pouches should not replace fresh, minimally processed foods and their use should be minimised beyond the complementary feeding period.^22^ The WHO European Network on Reducing Marketing Pressure on Children has highlighted the need for stricter regulations on the marketing of commercial baby foods, including pouches, due to their high sugar content and the risk of reinforcing poor eating behaviours.^15^ Additionally, public health guidelines in some countries recommend that young children be encouraged to eat from a spoon or hands rather than sucking from pouches, to support oral motor skill development and mindful eating habits.^23^

While many reviews have examined the poor nutritional composition of squeeze pouches,^3^, ^16^, ^17^, ^18^ little is known of the frequency and types consumed, sociodemographic characteristics of users, nor parental experiences around their use. Therefore, a broad synthesis of current evidence concerning their use through a scoping review is needed. Accordingly, the aim of this scoping review was to identify and collate the existing literature on the use of squeeze pouches among children aged 0–17 years, describing the frequency and types of squeeze pouches consumed, the sociodemographic characteristics of users and insights into parental experiences.

## 2 METHODS

This scoping review was guided by the Joanna Briggs Institute (JBI) methodology for scoping reviews. As per the JBI recommendations, a three‐step search strategy was utilised. The scoping review protocol was registered on Open Science Framework (https://doi.org/10.17605/OSF.IO/6X587). The PRISMA‐ScR Checklist was used to guide the reporting of this scoping review.

The review process began with an initial search of three online databases (MEDLINE, Scopus and CINAHL). This search was first conducted in January 2024 and was updated using refined search terms on the 2nd of April 2024, along with a grey literature search and reference list screening. The databases were queried using a combination of search terms related to participants and concepts, as detailed in Table 1. The search terms within each domain were searched individually using the Boolean operator ‘OR’ and then combined using the Boolean operator ‘AND.’ Next, a grey literature search was performed via Google Advanced Search using the same combination of search terms to identify primary research presented in published theses. Lastly, the reference lists of the identified articles and reports were screened to uncover additional studies. All search results were imported into EndNote (Version 20) for screening.

**TABLE 1:** Search strategy based on population, concept and context framework.

Search domain	Search terms
Participants: mothers, parents, caregivers and children (aged 0–17 years)	mother* OR parent* OR infant* OR child*
Concept: squeeze pouch	‘squeeze pouch*’ OR ‘food pouch*’ OR ‘baby food pouch’ OR ‘commercially packaged baby*’ OR ‘food, commercially packaged’ OR ‘complementary food’ OR ‘baby puree’ OR ‘yoghurt pouch*’ OR ‘fruit pouch*’ OR ‘fruit pouch consumption’ OR ‘pureed baby food*’ OR ‘plastic squeezable pouch*’
Context: location	N/A

Studies were eligible for inclusion if they addressed any of the following four key concepts: frequency, type, sociodemographic characteristics and parental experiences of squeeze pouch use. The frequency parameter specifically examined the consumption patterns of squeeze pouch use among children aged 0–17 years. This review included studies from all countries across a range of settings to capture all relevant evidence on the four key concepts pertaining to squeeze pouch use. Studies were not excluded based on study design and there was no limitation on publication date, therefore studies which met the inclusion criteria were included until April 2024. The participants included mothers, fathers, parents or non‐maternal caregivers with children aged between 0 and 17 years. The term ‘non‐maternal caregiver’ is defined a diverse group of caregivers such as fathers, grandparents or day care workers who have primary or secondary responsibilities of a child, or children.^24^

Following removal of duplicates, title and abstract screening, full‐text screening was undertaken by two reviewers independently using pre‐defined inclusion and exclusion criteria, structured according to the participants, concept and context criteria (see Table S1 for overview). Prior to title and abstract screening, a calibration exercise was conducted to ensure consistency between reviewers in applying the inclusion and exclusion criteria. For articles where there was a conflict between the two reviewers or where one reviewer considered it was ‘unclear’ whether it should be included, the article was screened by a third researcher.

Data extraction tables were developed for each of the four key concepts (frequency, type, sociodemographic characteristics and parental experiences), including details such as study setting, participant characteristics and outcomes. These tables aimed to extract dietary assessment methods utilised (e.g., interviews, food frequency questionnaires [FFQs], self‐report tools), the types of squeeze pouches consumed, sociodemographic characteristics associated with use and both quantitative and qualitative parental experiences. Data were extracted and charted iteratively in four predefined tables corresponding to the four key concepts, with categories refined as necessary during the synthesis process. All data were extracted by one author and checked for completeness and correctness by a second author. Following JBI guidelines, no formal quality assessment was conducted, as scoping reviews aim to map the existing literature rather than evaluate the methodological quality of included studies. Results were synthesised narratively, with quantitative data summarised using descriptive statistics and qualitative findings grouped thematically.

## 3 RESULTS

**FIGURE 1:**
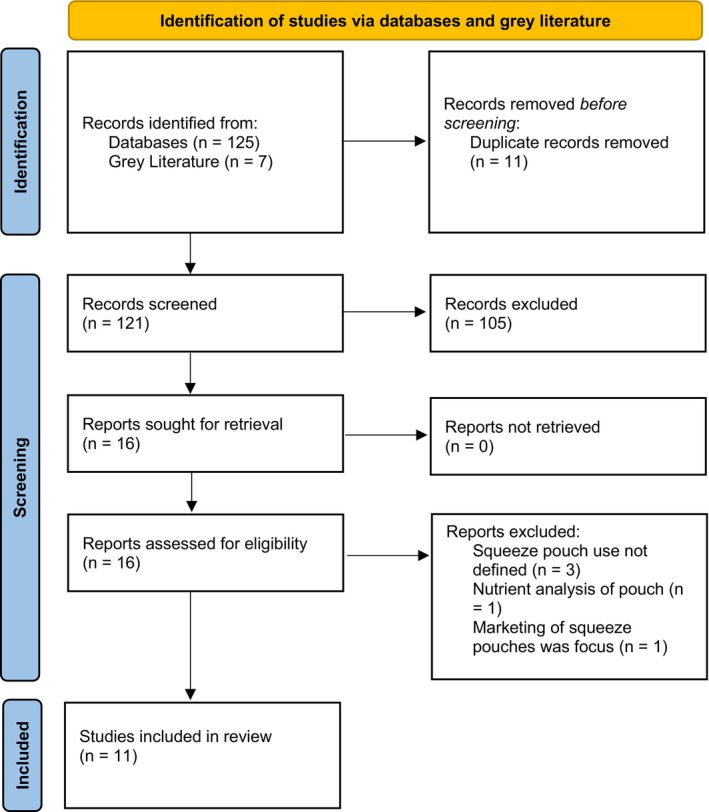
Preferred Reporting Items for Systematic Reviews and Meta‐Analyses extension for Scoping Reviews (PRISMA‐ScR) flow diagram representing search, screening and inclusion process.

As summarised in the PRISMA diagram (see Figure 1), title and abstract screening identified 125 articles, and the grey literature and the reference list search identified a further seven articles, with 121 remaining after duplicates were removed. Full text copies of each paper were obtained and stored in Endnote for review. From 121 studies, 16 full‐text articles met the inclusion criteria, and all 16 article full texts were screened against the inclusion criteria, after which 11 remained and 5 were excluded. Table 2 presents a high‐level summary of the included quantitative and qualitative articles against the key concepts relevant for this review: frequency of squeeze pouch use (*n* = 8),^25^, ^26^, ^27^, ^28^, ^29^, ^32^, ^33^, ^34^ type of squeeze pouches consumed (*n* = 3),^28^, ^29^, ^34^ sociodemographic characteristics associated with squeeze pouch use (*n* = 2),^28^, ^34^ and studies of parental experiences (*n* = 5).^10^, ^28^, ^30^, ^31^, ^32^, ^34^

**TABLE 2:** Current findings in the literature on squeeze pouches and link to key concept.

References	Setting	Sample size	Participant characteristics	Frequency	Type	Sociodemographic characteristics	Parental experience
Quantitative studies							
Cox et al.^25^	Carer‐infant dyads in Auckland and Dunedin, New Zealand	*n* = 625 Infant aged 6.5–9.5 months; 46.2% were female, 53.8% were male; 55% European, 21% Māori, 14.4% Asian, 7% Pacific, 2.6% other	98.7% of respondents were mothers; mean parental age 32.7 years; 64.9% of respondents held a university degree; 11.2% of respondents worked full‐time, 21.9% worked part‐time; 26.1% high socioeconomic deprivation, 45.1% middle socioeconomic deprivation, 28.8% low socioeconomic deprivation	√			
Fredriksson et al.^26^	Mother–child dyads in Västerbotten, Sweden	*n* = 2959 Infants aged 9 months	100% of respondents were mothers; mean maternal age 31.0 years; 68.6% of mothers held a university degree, 27.1% of mothers completed high school, 2.5% of mothers completed <9 years of school	√			
Lundkvist et al.^27^	Mother–child dyads in Västerbotten, Sweden	*n* = 1499 Children aged 18 months	100% of respondents were mothers; mean maternal age 30.9 years; 55.6% of respondents completed ≥3 years post‐secondary education	√			
Bruckner et al.^28^	Parents and/or carers‐infant dyads in Auckland and Dunedin, New Zealand	*n* = 300 Infants/children aged 6 months–3.9 years	95.8% of respondents were mothers; mean parental age 33.0 years; 53.9% New Zealand European, 19.2% Māori, 14% Pacific, 11.2% Asian, 1.8% other; 58.4% of respondents held a University degree; 21.8% of respondents worked full‐time, 31.2% worked part‐time, 35.1% were unemployed, 7.7% were on unpaid parental leave, 4.2% were on paid parental leave; 29.4% high socioeconomic deprivation, 43.3% middle socioeconomic deprivation, 27.3% low socioeconomic deprivation	√	√	√	√
Haszard et al.^29^	Parents and/or carers‐infant dyads in Auckland and Dunedin, New Zealand	*n* = 645 Infants/children 6–11.9 months; mean age 8.4 months; 46.1% were female, 53.8% were male; 54.6% European, 21.1% Māori, 14.1% Asian, 7.6% Pacific, 2.6% other; 66.1% were currently breastfed; 26.2% high socioeconomic deprivation, 44.7% middle socioeconomic deprivation, 29.2% low socioeconomic deprivation	Total sample demographics not presented	√	√		
Qualitative studies							
Rowan et al.^10^	Publicly available online forums	*n* = 6 (78 threads) Children aged 6–24 months	Total sample demographics not presented				√
Isaacs et al.^30^	Parents and/or carer‐infant dyads in England	*n* = 62 Infants aged 4–6 months	98.4% of respondents were mothers; 69.4% white British, 14.5% British, 8.1% white; 45% primiparous 6.5% single parent household				√
Neve et al.^31^	Parents and/or carer‐infant dyads in England	*n* = 62 Infants aged 4–18 months	98.4% of respondents were mothers; 93.% living in a multi‐parent household; 54.8% had at least two children residing in their household				√
Mixed‐methods							
Klerks et al.^32^	Parent‐infant dyads in four major cities in Spain (Madrid, Barcelona, Sevilla and Valencia).	*n* = 149 Infants aged 12–48 months; mean age 30.2 ± 11.0 months, 50.3% were female, 49.7% were male	83.9% of respondents were mothers, mean parental age 35.7 years	√			√
Iyer et al.^33^	Mother–child dyads in Western Sydney, New South Wales, Australia	(*n* = 29) Children aged 0–5 years; mean age 2 years; 51.7% were female, 48.8% were male; 6.9% identified as either Aboriginal and/or Torres Strait Islander	100% of respondents were mothers, mean maternal age 35 ± 3.6, 51.7% European, 20.7% Vietnamese, 13.8% Chinese, 6.9% mixed Asian, 3.4% Indonesian, 3.4% Aboriginal and/or Torres Strait Islander; 86.2% of respondents held a Diploma, degree or higher, 13.8% of respondents held a certificate or lower; 58.6% of respondents were in paid employment; 37.9% low socioeconomic status, 20.7% middle socioeconomic status, 41.4% high socioeconomic status	√			
McLean et al.^34^	Parents and/or carers‐infant dyads in Auckland and Dunedin, New Zealand	*n* = 625 Infants aged 7–10 months; 46.2% were female, 53.8% were male; 55% European, 21% Māori, 14.4% Asian, 7% Pacific, 2.6% other	98.7% of respondents were mothers; mean parental age 32.7 years; 64.9% of respondents held a university degree; 11.2% of respondents worked full‐time, 21.9% worked part‐time; 26.1% high socioeconomic deprivation, 45.1% middle socioeconomic deprivation, 28.8% low socioeconomic deprivation	√	√	√	√

Included studies were published between 2021 and 2024 and reported squeeze pouch use in children aged from 4 to 48 months. The included studies were conducted across five high‐income countries, including New Zealand (*n* = 4),^25^, ^28^, ^29^, ^34^ Sweden (*n* = 2),^26^, ^27^ the UK (*n* = 2)^30^, ^31^ and one each in Spain^32^ and Australia.^33^ No studies meeting the eligibility criteria were identified in middle‐ and low‐income countries. Most studies (*n* = 8) collected data on mother–child dyads directly.^25^, ^28^, ^29^, ^30^, ^31^, ^32^, ^33^, ^34^ Multiple publications reported on different outcomes using data from national surveys including the NorthPop study in Sweden,^26^, ^27^ First Foods New Zealand^25^, ^29^, ^34^ and The Young Foods New Zealand studies.^28^, ^29^ One study adopted a netnography approach,^10^ which is a qualitative research methodology that adapts ethnographic techniques to study online communities, consisting of six online forums in which 78 ‘threads’ were analysed.^10^ Five studies were quantitative in design,^25^, ^26^, ^27^, ^28^, ^29^ three were mixed methods,^32^, ^33^, ^34^ and three were qualitative.^10^, ^30^, ^31^ The sample size of the included studies ranged from *n* = 300 to *n* = 2959 for quantitative studies, *n* = 6 to *n* = 62 for qualitative studies and from *n* = 29 to *n* = 625 for mixed‐methods studies (Table 2).

An overview of the outcomes of included studies that reported on frequency‐related data is presented in Table 3. Of the included studies (*n* = 8), the frequency statistics were determined using a range of instruments and frequency was inconsistently defined, with studies variously reporting daily or weekly or monthly consumption, for example. The prevalence of squeeze pouch consumption (ever consumed) ranged from 23.5% to 85.4% of infants and children.^25^, ^26^, ^27^, ^28^, ^29^, ^32^, ^33^, ^34^ Most studies reported that infants and children consumed these foods frequently: in line with each study's measurement interval, between 8.7% and 29.2% of infants and children consume squeeze pouches daily,^28^, ^32^, ^33^, ^34^ 20.9%–75.2% weekly,^25^, ^26^, ^27^, ^28^, ^32^, ^33^, ^34^ and 16.7%–70.4% consume them monthly.^25^, ^26^, ^27^, ^28^, ^32^, ^33^, ^34^ One study reported that squeeze pouch consumption was associated with poor eating behaviours such as ‘food responsiveness,’ ‘food fussiness’ and ‘selective/restrictive eating.’^25^ Other studies found that between 16.7% and 47.8% of infants had previously consumed food from a squeeze pouch prior to the recommended 6 months of age.^33^, ^34^ Additionally, some studies reported on how the contents of squeeze pouches were consumed, with one study reporting that 65% of toddlers and preschool aged children sucked directly from the nozzle.^28^ However, another study found that of the infants who consumed foods from squeeze pouches frequently (five or more times per week; 28%), only 5% consumed directly from the nozzle.^34^

**TABLE 3:** Overview of included studies that reported on the prevalence and frequency of squeeze pouch use.

References	Method of data collection	Squeeze pouch outcome measure	Analysis method used	Interpretation of results or key findings relating to key concepts
Cox et al.^25^	Interview; in‐person; First Foods New Zealand	24‐h dietary recalls, self‐administered electronic questionnaires; Child Eating Behaviour Questionnaire: a 35‐item questionnaire; Paediatric Eating Assessment Tool: a 78‐item questionnaire	Descriptive statistics	28% of infants consumed food from pouches frequently (≥5 times per week during the past month) Pouch consumption was associated with greater ‘food responsiveness,’ ‘food fussiness’ and ‘selective/restrictive eating’
Fredriksson et al.^26^	Online survey; Northpop catchment area	Self‐administered food frequency questionnaire	Descriptive statistics	80.7% of infants consumed fruit pouches ranging from rarely (one to three times per month) to very frequently (four to six times per day) 51.5% of infants consumed one or more fruit pouches per week
Lundkvist et al.^27^	Online survey; Northpop catchment area	Self‐administered 1‐month recall food frequency questionnaire	One‐way ANOVA, chi‐square analysis	76.5% of children were non‐consumers of fruit pouches 23.5% of children consumed fruit pouches once or more per week
Bruckner et al.^28^	Interview; in‐person; The Young Foods New Zealand Photo‐elicitation exercise	24‐h recalls, self‐administered electronic food frequency questionnaire	Descriptive statistics, one‐sample *t*‐tests	85.4% of children had consumed a squeeze pouch at some point in their life Of this: 38% of children were aged 12–24 months 62% of children were aged 2–4 years 61.7% of children consumed a squeeze pouch ≤1 per month 8.7% of children consumed a squeeze pouch two to three times per month 8.0% of children consumed a squeeze pouch 1 time per week 10.5% of children consumed a squeeze pouch two to four times per week 2.4% of children consumed a squeeze pouch five to six times per week 8.7% of children consumed a squeeze pouch ≥1 per day Overall, 11% of children consumed a squeeze pouch frequently (five or more times per week) 65% of all toddlers and preschool aged children consumed food from a squeeze pouch directly from the nozzle
McLean et al.^34^	Interview; in‐person; First Foods New Zealand Online surveys	Interviewer‐administered 24‐h recalls; self‐administered electronic questionnaire	Descriptive statistics, logistic regression models	78.7% of infants consumed a squeeze pouch on at least one occasion prior to the study 26.7% of infants were consuming at least some food from a squeeze pouch when solids were introduced 47.8% of infants consumed a squeeze pouch by 6 months of age 28% of infants were frequent squeeze pouch consumers (five or more times per week) The median frequency of squeeze pouch use in all participants was one per week The median frequency among frequent squeeze pouch consumers was seven times per week In the time period of 1 month, overall, 20% had consumed a squeeze pouch daily, 33% weekly and 18% monthly In the time period of 1 month, in infants aged 6 months, 13% had consumed a squeeze pouch daily, 23% weekly and 10% monthly The proportion of food occasionally consumed from a squeeze pouch directly from the nozzle differed among age groups, 42% at age of assessment (mean age 8.4 months), 26% at 6 months and 16% when first introduced to solids For infants who consumed squeeze pouches frequently, the likelihood of consuming foods from squeeze pouches directly from the nozzle was 5% of all infants at their current age, 2% at 6 months of age and 0.5% when first introduced to solids
Klerks et al.^32^	Interview; in‐person	Interviewer‐administered survey via hedonic scale	Descriptive statistics, one‐sample *t*‐tests	10.7% of toddlers consumed a yoghurt pouch every day 23.5% of toddlers consumed a yoghurt pouch 2–4 days per week 51.7% of toddlers consumed a yoghurt pouch 1–2 days per week 66.4% of toddlers were given a yoghurt pouch during mid‐afternoon
Iyer et al.^33^	Semi‐structured interviews via phone Online survey	Interviewer‐administered survey, self‐administered electronic questionnaires, 24‐h recalls; Automated Self‐Administered 24‐h Dietary Assessment Tool (ASA24)	Descriptive statistics, Shapiro–Wilk test, ANOVA, chi‐square analysis	82.8% of children had consumed a squeeze pouch From this: 29.2% of children used squeeze pouches at least once daily 25% of children used squeeze pouches at least once weekly 16.7% of children used squeeze pouches at least twice monthly 16.7% of children were introduced to squeeze pouches at <6 months of age 37.5% of children were introduced to squeeze pouches at 6 months of age 41.7% of children were introduced to squeeze pouches at >6 months of age
Haszard et al.^29^	Interview; in‐person; First Foods New Zealand and The Young Foods New Zealand Photo‐elicitation exercise Online survey	Interviewer‐administered 24‐h recalls; self‐administered electronic questionnaire	Descriptive statistics	45.3% of infants consumed food from a squeeze pouch on ≥1 of the 2 recall days Across the 2 recall days, infants who consumed squeeze pouches, did so on a mean (SD) of 1.3 (0.9) occasions per day On recall days where squeeze pouches were consumed, the mean (SD) was 1.6 (0.8) occasions per day

Various methods were used to determine the frequency of squeeze pouch consumption. For example, some studies measured frequency using self‐reported^25^, ^26^, ^27^, ^28^, ^29^, ^33^, ^34^ or interviewer administered questionnaires.^32^ Five studies measured the frequency of squeeze pouch consumption using various dietary assessment tools,^25^, ^28^, ^29^, ^33^, ^34^ including an Automated Self‐Administered 24‐h Dietary Assessment Tool (ASA24),^33^ Child Eating Behaviour Questionnaire and the Paediatric Eating Assessment Tool.^25^ The recall periods for these tools ranged from ‘daily’ to ‘monthly.’ One study quantified the dietary contribution of squeeze pouches. Using 24‐h recall data, they reported that pouches provided 25.5% of total energy from complementary foods among infants who consumed them on the recall day, and 11% across all infants in the sample.^29^ No other included studies reported the percentage of total dietary energy or nutrient intake attributable to squeeze pouches.

An overview of the outcomes of included studies that reported data on the type of squeeze pouch is presented in Table 4. Studies (*n* = 3) utilised self‐administered electronic questionnaires and interviewer‐administered 24‐h recalls to gain data on the types of squeeze pouches consumed.^28^, ^29^, ^34^ Three studies examined the types of squeeze pouches consumed using self‐administered electronic questionnaires and interviewer‐administered 24‐h recalls.^28^, ^29^, ^34^ Two studies categorised pouches as ‘dairy‐based,’ ‘fruit‐based,’ ‘vegetable‐based,’ ‘meat and fish‐based,’ ‘breakfast cereal‐based,’ ‘cereal/grain/pasta‐based,’ or ‘unknown/mixed,’^28^, ^34^ while another used the Ministry for Primary Industries‐University of Otago food group system.^29^

**TABLE 4:** Overview of included studies that reported on the type of squeeze pouch used, sociodemographic characteristics of study participants categorised as squeeze pouch users, and the parental experience when using squeeze pouches.

References	Method of data collection	Squeeze pouch outcome measure	Analysis method used	Interpretation of results or key findings relating to key concepts
Types of squeeze pouches used				
Bruckner et al.^28^	Interview; in‐person; The Young Foods New Zealand Photo‐elicitation exercise	Interviewer‐administered 24‐h recalls, self‐administered electronic food frequency questionnaire	Descriptive statistics; one‐sample *t*‐tests	Most common squeeze pouch types among users: 43.3% dairy‐based 22.9% unknown 17.8% fruit‐based 4.8% vegetable‐based 4.8% meat and fish‐based 1.6% breakfast cereal‐based 0.6% cereal/grain/pasta‐based
McLean et al.^34^	Interview; in‐person; First Foods New Zealand Online surveys	Interviewer‐administered 24‐h recalls; self‐administered electronic questionnaire	Descriptive statistics; logistic regression models	Most common pouch types among users: 30.8% meat and fish‐based 28.2% fruit‐based 22.0% vegetable‐based 11.6% dairy‐based 5.0% breakfast cereal‐based 2.1% mixture of squeeze pouch types 0.2% cereal/grain/pasta‐based
Haszard et al.^29^	Interview; in‐person; First Foods New Zealand and The Young Foods New Zealand Photo‐elicitation exercise Online survey	Interviewer‐administered 24‐h recalls; self‐administered electronic questionnaire	Descriptive statistics	Most common squeeze pouches among users: 45.3% all pouches 24.2% fruit or vegetable‐only 17.4% fruit‐based 9.2% dairy‐based 7.1% fruit and vegetable‐combined
Sociodemographic characteristics of frequent users				
Brucker et al.^28^	Interview; in‐person; The Young Foods New Zealand Photo‐elicitation exercise	Self‐administered electronic questionnaire	Descriptive statistics	Broadly, there were only minimal difference between frequent pouch use (five or more times per week) and non‐frequent pouch use (at least once in the past month but fewer than five times per week). Of the frequent pouch users: 46.9% were of New Zealand European ethnicity and 25.0% were of Māori ethnicity 53.1% held a university degree or higher 37.5% were not currently employed 34.4% were employed full‐time 59.4% had an area level deprivation score of 4 to 7 (middle socioeconomic deprivation) 37.5% had two children usually residing in their household (including study child) 53.1% used preschool/childcare
McLean et al.^34^	Interview; in‐person; First Foods New Zealand Online surveys	Self‐administered electronic questionnaire	Descriptive statistics; logistic regression models	Compared to non‐pouch users, frequent pouch users (five or more times per week) were: 4.7 times more likely to be Māori (OR [95% CI] 4.72 [2.51, 8.87]) 2.6 times more likely to be Pacific Islander (OR [95% CI] 2.55 [1.07, 6.13]) 4.7 times more likely to have a primary or secondary education level (OR [95% CI] 4.72 [2.48, 8.99]) 3.2 times more likely with a polytechnic or similar tertiary education level (OR [95% CI] 3.15 [1.70, 5.85]) 3.9 times more likely to be obese (≥30 kg/m^2^) (OR [95% CI] 3.85 [2.12, 6.97]) 2.5 times more likely to attend childcare or home‐based care (OR [95% CI] 2.46 [1.26, 4.80]) 3.2 times more likely with three or more children were living in the household (OR [95% CI] 3.16 [1.74, 5.73]) 2.5 times more likely to have an area level deprivation score of 8 to 10 (high socioeconomic deprivation) (OR [95% CI] 2.54 [1.41, 4.58]) 3.3 times more likely if the duration of exclusive breastfeeding was <1 month (OR [95% CI] 3.28 [1.92–5.60]) 4.7 times more likely if the duration of exclusive breastfeeding was 1–4 months (OR [95% CI] 4.67 [2.51–8.68]) 9.7 times more likely if the age when solids were introduced was <5 months (OR [95% CI] 9.67 [4.81–19.45]) 2.0 times more likely if the age when solids were introduced was 5 months (OR [95% CI] 1.99 [1.18–3.36])
Parental experiences using squeeze pouches				
Klerks et al.^32^	Interview; in‐person	Interviewer‐administered survey via hedonic scale		Lower levels of squeeze pouch acceptability were motivated by the addition of sugar: ‘It [yoghurt pouch] has sugar and I just don't like my child's diet containing sugar,’ ‘It [yoghurt pouch] has a lot of sugar and little fruit,’ ‘Some ingredients are unnatural and not acceptable for the age the product is targeted to.’ High levels of squeeze pouch acceptability were found among parents who positively valued the naturalness of the product and the elimination of sugar: ‘The best thing is that it does not have preservatives or additives, it is a natural product,’ ‘It is a natural product, with no sugar.’
Rowan et al.^10^	Netnographic analysis; Internet‐based communications on publicly available online forums	Threads relating to squeezable baby food pouches	Thematic analysis	Perceptions of baby food pouches fell into 2 broad categories: ‘benefits’ (*n* = 628) and ‘concerns’ (*n* = 337) The most common reported themes related to benefits were convenience, health, baby enjoys, variety and cost. The most common reported concerns were health, cost, lack of dietary diversity, dependence and waste.
Isaacs et al.^30^	Semi‐structured interviews; via phone or video conference Photo‐elicitation exercise Online survey	Self‐administered electronic questionnaire	Reflexive thematic analysis	Convenience Purees feel safe for parents who are anxious about feeding and packaging reinforces this Purees were often considered to be a cost‐effective option Brand eco‐systems provide reassurance
Brucker et al.^28^	Interview; in‐person; The Young Foods New Zealand	24‐h recalls, self‐administered electronic food frequency questionnaire		The most common reported themes relating to squeeze pouch use were convenience, health, variety, child enjoys, freshness, cost and ‘have heard good things about them.’ The most common reported themes relating to dislikes were health, environmental impacts, unknown ingredients, preference for homemade meals, freshness, cost, portion size, quality, texture, lack of variety, lack of control, requires effort to clean, dislikes nozzle, child does not enjoy, child may prefer them to real food and everything
McLean et al.^34^	Interview; in‐person; First Foods New Zealand Online surveys	Interviewer‐administered 24‐h recalls; self‐administered electronic questionnaire		Of pouch users (of any duration), most (90%) reported that ‘convenience’ was one of the reasons they used squeeze pouches, with pouches being described as easy to use (63%), practical (53%) and takes less time (36%). Other reasons for using squeeze pouches included ‘baby enjoys’ (35%), for ‘health’ reasons (33%) (in which nutrition (29%), easy to get fruits and vegetables in (20%) and easy to get meat in (17%) were reported as reasoning) and to increase the ‘variety’ of food their child eats (24%). Of all participants (including pouch users of any duration and non‐pouch users), there was a higher proportion of non‐pouch users (81%) reporting dislikes compared to pouch users (57%).
				Of all participants, more than a quarter (26%) reported that ‘health concerns’ was one of the reasons they dislike squeeze pouches, which was higher in non‐pouch users compared to pouch users (38% vs. 23%, respectively). Other reasons for disliking squeeze pouches included environmental concerns (17%), having other food preferences (13%) and a lack of information on what was in the squeeze pouches, or preparation methods (11%)
Neve et al.^31^	Semi‐structured interviews; via phone or video conference Photo‐elicitation exercise	Self‐administered electronic questionnaire	Reflexive thematic analysis	Common reported themes reported by parents included: Convenient to transport and store in a bag Trust in branding and front‐of‐pack ‘claims’ Reassurance that their child was consuming fruits and/or vegetables when fresh fruits and vegetables were refused: *‘That's why I like the pouches as well, because he won't eat that much fruit and vegetables that I put in front of him*. *If I can still give him a pouch every day then he's getting some sort of … fruits and vegetables.’* Perceived health benefits from claims made by brand Useful in time‐poor situations: *‘If [infant] has a convenience meal, then it's usually a night when either I'm out or we've decided to have a takeaway later on in the evening. Or if I'm just short on time, we'll give him his dinner first and do his bedtime routine, and then we'll have our dinner later on.’* Squeeze pouches were an option when parents were uncertain about what to feed their child, or lacked confidence: *‘Ella's Kitchen pouches, I've been trying him with because I can see all the ingredients. I don't have the confidence or the time, really, to make stuff myself.’* Fathers were more likely to provide squeeze pouches due to lack of confidence in what to feed their children: *‘My partner will only really give [child] something very easy. He would never look in a recipe book or make him a recipe that would always be what I do*.’

Findings varied by age group. Among children aged 6 months to 3.9 years, dairy‐based pouches were most common (43.3%), followed by fruit‐based (17.8%) and both vegetable and meat/fish‐based (4.8%).^28^ In infants aged 6–11.9 months (mean age 8.4 months), fruit‐based and fruit/vegetable‐only pouches were most used (41.6%), with dairy‐based at 9.2%.^29^ In infants aged 7–10 months, meat and fish‐based pouches were most consumed (30.8%), followed by fruit‐based (28.2%), vegetable‐based (22.0%) and dairy‐based (11.6%).^34^

An overview of the outcomes of the two included studies that reported sociodemographic‐related data of squeeze pouch users is presented in Table 4. Sociodemographic data were obtained through self‐administered electronic questionnaires.^28^, ^34^ One study examined the proportion of various demographic groups who were frequent squeeze pouch users (five or more times per week) and reported that 47% of the participants were New Zealand European, and 25% were Māori; however, this study did not conduct hypothesis testing to determine statistically significant differences between demographic groups.^28^ The other study used logistic regression models and found that when compared to non‐pouch users, frequent pouch users were 4.7 times more likely to be Māori, and 2.6 times more likely to be Pacific Islander.^34^ There was no association for participants with European or Asian ethnicity.^34^ Education was considered by both studies, showing mixed results. One study reported that of frequent pouch users, 53% held a university degree or higher qualification compared to participants who held either a polytechnic (21.9%) or school level education (25%).^28^ Conversely, the other study which conducted hypothesis testing reported that, compared to those with a university degree or higher qualification, participants with primary or secondary education levels were 4.7 times more likely, and participants with a polytechnic education were 3.2 times more likely to be frequent pouch users.^34^

Employment factors did not affect the likelihood of being a frequent pouch user in either study. For example, frequent pouch use was seen in participants from various employment backgrounds, notably from both unemployed and full‐time employed (37.5% and 34.4% of frequent squeeze pouch users, respectively).^34^ Further, there was no significant association between employment status and frequent pouch use.^28^ Area‐level socioeconomic status was also explored. One study reported that frequent pouch users were more likely to come from areas of middle to high socioeconomic deprivation,^28^ and similarly, the other study reported households in high area level deprivation were 2.5 times more likely to be frequent squeeze pouch users.^34^

Household composition was reported to impact the frequency of squeeze pouch use. For example, in households where two children (including the study child) were typically present, 37.5% exhibited frequent pouch use,^28^ whereas infants from households with three or more children had increased odds of frequent pouch use compared to those from single‐child households.^34^ Similarly, frequent pouch use was more common in households where children went to childcare,^28^ with one study reporting a 2.5‐fold increase compared to no use of formal childcare.^34^ Squeeze pouch use was also associated with other complementary feeding practices. For example, frequent squeeze pouch use was significantly higher if infants ceased breastfeeding at <1 month (3.3 times), between 1 and 4 months (4.7 times) or if the age when infants were introduced to solids was <5 months (9.7 times) or at 5 months (2.0 times) compared to at age 6 months.^34^

An overview of the included studies that reported data on the parental experience when using squeeze pouches is presented in Table 4. Data pertaining to the parental experience were obtained mostly through interviews; either in person (*n* = 3)^28^, ^32^, ^34^ or via phone or video conference with an added photo‐elicitation exercise (*n* = 2).^30^, ^31^ One study utilised a netnographic analysis of ‘threads’ from internet‐based communications on publicly available online forums.^10^ Notably, 83% (*n* = 5) of the studies examining parental experiences found that ‘convenience’ was one of the main benefits of using squeeze pouches,^10^, ^28^, ^30^, ^31^, ^34^ with one study describing squeeze pouches as being ‘easy to use,’ ‘practical’ and ‘takes less time.’^29^, ^34^ Other common themes identified with squeeze pouch use pertained to ‘health,’^10^, ^28^, ^31^, ^32^, ^34^ ‘variety,’^10^, ^28^, ^34^ ‘baby enjoys’^10^, ^28^, ^34^ and ‘cost.’^10^, ^28^, ^30^, ^34^ Two thirds of the included studies (67%) examined concerns faced by parents,^10^, ^28^, ^32^, ^34^ highlighting prevalent themes such as ‘waste/environmental impacts,’ ‘cost,’ ‘lack of diversity’ and ‘health/ingredients.’ One study reported that there was a higher percentage of non‐pouch users (81%) who reported concerns about pouch use compared to pouch users (57%).^34^

## 4 DISCUSSION

To the authors' knowledge, this scoping review is the first to explore the existing literature on foods in squeeze pouches, including the frequency of consumption, the types of pouches consumed, the sociodemographic characteristics associated with squeeze pouch users and insights from parental experiences for children aged 0–17 years. Most studies reported that a substantial proportion of infants and children between the ages of 6 months and 5 years were regularly eating these products,^25^, ^26^, ^27^, ^28^, ^32^, ^33^, ^34^ particularly fruit and dairy‐based squeeze pouches as part of their diet.^28^, ^29^ The high prevalence of parents reporting using these products is unsurprising given the recent transformation of the international commercial infant food market, in which squeeze pouches have a dominant and growing market share.^18^

In terms of drivers of common and frequent food pouch consumption, infant food manufacturers have developed squeeze pouches for their convenience and portability, often promoting them as a ‘mess‐free’ alternative to other complementary feeding methods, which may have shaped societal norms regarding product desirability and acceptance.^35^ Concurrently, wider social trends, such as time constraints faced by caregivers, may influence feeding practices and contribute to the increasing use of convenience‐based food products like squeeze pouches.^36^, ^37^ Further, child feeding practices are also shaped by parental beliefs, values and knowledge, in particular around the healthiness of foods; therefore, the marketing of squeeze pouches as healthy and age‐appropriate for infants as young as 4 months, and older children may also explain the high consumption of these products.^38^, ^39^

Despite the marketing of these products as healthy, recent studies from several countries have highlighted the poor nutritional composition of these products, with significant concerns expressed by relevant expert groups regarding their high sugar content, low iron and potential oral concerns and risk of obesity.^1^, ^40^ These findings highlight the need for further research to better understand who is using these products and how consumption patterns vary across populations, which could help inform future public health discussions.^15^ It also raises important questions about the potential health implications both for infants and older children and adolescents, which remain largely unexplored in current research. Since completing this review, a new large‐scale observational study by McLean et al.^41^ has provided further insight into the frequency of pouch use and associated outcomes in a diverse sample of New Zealand infants, toddlers and preschoolers.^41^ This study found that while squeeze pouch use was common, frequent use declined with age and relatively few children both frequently used pouches and consumed them directly from the nozzle. Importantly, there was no association between pouch use and BMI *z*‐scores in any age group. Interestingly, infants who used pouches had slightly higher energy intake from complementary foods, while preschool‐aged children who were frequent users had lower total energy intake compared to non‐users. These findings suggest that concerns about excessive energy intake and weight gain related to pouch use may not be substantiated by current evidence, though longer‐term impacts and dietary quality require further investigation.

Regarding the type of squeeze pouches consumed, our review has identified the use of predominantly fruit and dairy‐based squeeze pouches in the literature. However, the studies reporting type‐related data were conducted exclusively in New Zealand. Therefore, the preference for these specific pouches could be related to their varying availability internationally, or due to the fact that most commercial infant foods are largely fruit‐based. Wider preferences may also be related to parents' own dietary preferences and perceived healthiness of these foods.^17^ For example, dairy‐based foods are perceived by parents as providing essential nutrients like calcium and protein for growing children.^42^ However, the high use of these types of products is concerning, as global nutrient analyses show that fruit‐based and dairy‐based squeeze pouches contain higher added sugar content.^1^, ^3^, ^18^ Infants have a natural preference for sweet tastes, and some studies suggest that early exposure to high‐sugar foods may influence taste preferences.^1^, ^15^ However, further research is needed to understand the long‐term impact of squeeze pouch consumption on dietary habits and health outcomes.^43^, ^44^ Additionally, while frequency of intake was commonly reported, only one study quantified the extent to which pouches contributed to dietary intake finding that pouches form a substantial component of some infants' diets, reinforcing concerns that these products may displace more nutrient‐dense foods during key stages of development. In older children, the convenience of squeeze pouches may contribute to more frequent snacking. However, further research is needed to understand whether this impacts structured mealtimes, hunger regulation or overall dietary patterns. The differences in nutritional profile of squeeze pouch foods developed for infant meals during the complimentary feeding period, in addition to an audit of ‘snack like’ squeeze pouch products developed for older children, warrants further exploration. Further research into the nutritional composition of squeeze pouches and their role in children's diets may help inform discussions on appropriate marketing and labelling guidelines. Such measures would help ensure that squeeze pouch foods intended for infants and young children align with age‐appropriate feeding recommendations and support healthy eating behaviours for older children that align with national dietary guidelines. To achieve this, further investigation into the impact of squeeze pouch consumption at different stages of infancy and childhood on food behaviour and health outcomes is warranted.

Our review has also highlighted the relative paucity of sociodemographic data available on squeeze pouch users, indicating a need for further research to better understand the diverse factors influencing squeeze pouch usage across different populations. The studies that did examine differences in squeeze pouch use by sociodemographic characteristics were both on New Zealand infants and parents, which may limit generalisability when examining broader populations. These studies presented conflicting results about the relationship between education and ethnicity and frequent squeeze pouch use, no relationship with parental employment and higher squeeze pouch use among residents of areas with higher socioeconomic deprivation. Wider research on infant and child feeding has consistently shown that socioeconomic factors play a role in shaping parental behaviours related to child feeding practices, particularly for families of lower socioeconomic status.^45^ For example, children of low socioeconomic status are at higher risk of consuming nutrient‐poor diets, lacking in whole fruits and vegetables.^46^ Additionally, families on lower incomes may face barriers to accessing or affording healthy food options, and therefore they may be more inclined to rely on convenience foods like squeeze pouches as an affordable feeding solution for their infants. However, the small number of studies and the mixed results within them mean there is a pressing need for further research that examines the relationship between sociodemographic characteristics and squeeze pouch use, particularly given the effect that these characteristics have on infant and child feeding practices and infant and child nutrition generally.^47^, ^48^ Further, given the high frequency of squeeze pouch use across sociodemographic groups, investigating parental perceptions of squeeze pouches is essential for developing education and advocacy that addresses the underlying motivators and enablers of their use.

The studies reviewed that examined parental perceptions found that parents view squeeze pouches as a convenient option for feeding their children due to factors such as portability, cost, minimal mess and ease of use. This is supported by broader complementary feeding literature which shows, despite infant feeding recommendations, parents often adopt inappropriate complementary feeding practices based on affordability and convenience.^11^, ^37^, ^49^ In some studies, parents expressed mixed views regarding the perceived health and cost of squeeze pouches; while some parents viewed them as beneficial, others expressed concerns about these aspects.^10^, ^28^, ^30^, ^31^, ^34^ In one study, parents reported being reassured that vegetable‐based squeeze pouches offered an alternative way to provide servings of vegetables to their children.^10^ However, due to a lack of industry regulation, commercially available squeeze pouches may claim vegetables as the primary ingredient when this is not often the case, as most vegetable‐based squeeze pouches are blended with free sugars, such as fruit puree, fruit concentrate and fruit juices.^1^, ^50^ Consequently, parents may need clearer advice about the potential negative impacts of relying on squeeze pouches for children's nutrition, including deficiencies in essential nutrients crucial to growth and development, and changes to their long‐term taste and eating preferences.^51^

The findings of this review have several implications for both research and practice. In terms of research, there is a clear need for studies across contexts that examine relationships between sociodemographic characteristics and the frequency and nature of squeeze pouch use so we can understand who is using them, which types and why. Additionally, based on market share information, it is likely that squeeze pouches are increasingly marketed to and consumed by school‐aged children and adolescents; thus, research is needed to understand consumption frequency and patterns across ages. Finally, as squeeze pouches are a relatively new product type and the body of literature examining them is in its early stages, longer‐term research is needed to examine their impacts on infant and child eating behaviour and nutrition. In terms of practical implications, the findings of this review that squeeze pouch use is common, often frequent, and dominated by sugar‐heavy fruit and dairy types can be used to substantiate public health concerns around the dietary intake received from squeeze pouches across key growth periods for children. The findings can inform the development of nutrition intervention through policy to better protect vulnerable populations and ensure all children have sufficient access to a quality diet to meet their nutritional requirements for optimal growth and development. Additionally, involving key health practitioners in the complementary feeding process by providing evidence‐based recommendations may provide invaluable support to parents, facilitating informed decision‐making and promoting optimal feeding practices for infants and older children. This review also comes at a significant moment in policy development in Australia and New Zealand.^52^ In 2025, food ministers endorsed a new policy position to guide approaches for improving commercial foods for infants and young children. This policy recognises the important role these foods play in growth, development and the establishment of long‐term eating patterns. Regulatory approaches have now been referred to Food Standards Australia New Zealand, and non‐regulatory approaches are being progressed by the Australian Government Department of Health and Aged Care in consultation with the Healthy Food Partnership. This ongoing policy work highlights the relevance of our review in providing timely insights into the types of products consumed, usage patterns and parental perceptions—critical considerations for ensuring that future regulations and public health efforts are aligned with infant and child nutrition needs.

A notable strength of this review is its comprehensive scope, inclusive of a diverse range of studies, methodologies and outcomes examining squeeze pouch use. However, our review has some limitations, including challenges synthesising studies due to methodological inconsistencies between studies. For example, in this review, the definition of ‘frequent’ squeeze pouch usage varied between studies, with some defining it as daily consumption, and others considering it as several times a week or month, making it challenging to draw direct comparisons. Therefore, the wide range of reported frequencies of intake could relate to these methodological differences or could be due to true differences in the availability of these products and perceptions of them in different regions and populations across the world. Future research should utilise standardised methodologies to quantify intake of squeeze pouches and in exploring parental experiences and perceptions of squeeze pouch usage. Additionally, our review identified only limited information regarding the percentage of total dietary intake from different types of squeeze pouches to an infant or child's overall dietary intake. Further, no data were available for older children or for total daily energy and nutrient intake, limiting our ability to understand the overall dietary role of these products across age groups. Similarly, the potential variability of the typical diet along with the overall nutrient intake among infants and children in different settings was not assessed in this review. This hinders our ability to accurately determine and predict squeeze pouch consumption, which is an important avenue for future research. Where possible, it could be recommended to report the contribution percentage of squeeze pouches to overall nutritional intake to streamline comparisons between studies and provide more accurate patterns of consumption. Similarly, where possible, we have described squeeze pouch usage by age group. However, inconsistencies in age reporting across studies limited comparisons. Future research should use standardised age categories and report prevalence and frequency consistently. For example, age groupings such as 6–11, 12–23, 24–36 and 36+ months may be more appropriate in measuring the consumption of squeeze pouches, as these ages reflect key developmental stages and are consistent with WHO guidelines for infant feeding.^53^ In addition, future studies should consider reporting usage among older age groups, including preschool‐ and school‐aged children, to capture broader patterns of use beyond infancy and toddlerhood. Further, few studies provided detailed information on whether children consumed the contents directly from the spout or were spoon‐fed. This distinction is important, as direct sucking from pouches may impact oral motor skill development and eating behaviours.^54^, ^55^ Given this gap in the literature, future research should explore feeding methods and their potential implications for child development. More generally, despite the large and growing use of squeeze pouches, the relatively few studies on their use make it difficult to derive clear and consistent findings and recommendations.

In conclusion, this scoping review highlights the widespread use of squeeze pouches, particularly those containing fruit and dairy, among infants and children, driven by factors such as convenience, cost and perceived healthiness. However, concerns regarding their nutritional composition and potential health implications highlight the need for further research to inform public health recommendations. It is recommended that future research adopt standardised methodologies to quantify intake of squeeze pouches and explore parental perceptions and predictors of usage to better understand patterns of consumption and their impact on child health. Future research examining the nutritional quality of squeeze pouches and their impact on child feeding practices may provide valuable insights for public health recommendations and consumer education. Further research should focus on identifying barriers to safe and nutritious feeding practices and wider data on squeeze pouch consumption in older children. This could be used to develop targeted education programs to promote optimal feeding practices that minimise the use of squeeze pouches in infants and older children.

## AUTHOR CONTRIBUTIONS

CAKF, KK, AS designed this scoping review protocol. BS conducted the database search. Screening and data extraction was led by BS with support from CAKF and KK. BS synthesised the extracted results and drafted the first version of the manuscript paper. All authors contributed to writing and editing the final manuscript.

## CONFLICT OF INTEREST STATEMENT

The authors declare no conflict of interest.

## Supporting information


**Table S1.** Inclusion criteria based on population, concept and context (PCC) framework.

## Data Availability

The data that support the findings of this study are available from the corresponding author upon reasonable request.
