# Supplementary material for: A scoping review of squeeze pouch use among infants and children: Frequency, types, sociodemographic characteristics and parental experiences
Source: Nutr Diet. 2025 Jul 24;83(1):125–44. doi: 10.1111/1747-0080.70030 (PMC12884246; doi:10.1111/1747-0080.70030)
Supplement: Supplementary file 1 — Table S1. Inclusion criteria based on population, concept and context (PCC) framework. [file NDI-83-125-s001.docx]

**Supplementary Table**

Supplementary Table 1: Inclusion Criteria Based on Population, Concept, and Context (PCC) Framework

Criteria

|  | Inclusion Criteria | Included |
| --- | --- | --- |
| Participants | Infants: aged 0 – 5 months  Early childhood: 6 months – 4 years  Middle childhood: 5 – 9 years  Adolescents: 10 – 18 years  Adults: aged above 18 years  Parents and/or caregivers of children aged between 0 –18 years | Yes  Yes  Yes  Yes  No  Yes |
| Concept | Frequency (i.e. how often squeeze pouch is consumed)  Type (e.g. fruit-based, dairy-based etc.)  Socio-demographics (e.g. education, income, socioeconomic status etc.)  Experience of parent (i.e. description of experiences or perceptions)  The nutritional composition of squeeze pouches  Breastfeeding  Baby-led weaning  Traditional spoon-fed  Complementary feeding (without the use of commercial squeeze pouches)  Marketing (of squeeze pouches) | Yes  Yes  Yes  Yes  No  No  No  No  No  No  No |
| Context | All studies  All settings  Studies published in foreign languages (other than English)   Full text not available | Yes   Yes  No   No |
